# An Improved TransMVSNet Algorithm for Three-Dimensional Reconstruction in the Unmanned Aerial Vehicle Remote Sensing Domain

**DOI:** 10.3390/s24072064

**Published:** 2024-03-23

**Authors:** Jiawei Teng, Haijiang Sun, Peixun Liu, Shan Jiang

**Affiliations:** Changchun Institute of Optics, Fine Mechanics and Physics (CIOMP), Chinese Academy of Sciences, Changchun 130033, China; tengjiawei@ciomp.ac.cn (J.T.); jiangs@ciomp.ac.cn (S.J.)

**Keywords:** reconstruction, deep learning, drone remote sensing, TransMVSNet, artificial intelligence

## Abstract

It is important to achieve the 3D reconstruction of UAV remote sensing images in deep learning-based multi-view stereo (MVS) vision. The lack of obvious texture features and detailed edges in UAV remote sensing images leads to inaccurate feature point matching or depth estimation. To address this problem, this study improves the TransMVSNet algorithm in the field of 3D reconstruction by optimizing its feature extraction network and costumed body depth prediction network. The improvement is mainly achieved by extracting features with the Asymptotic Pyramidal Network (AFPN) and assigning weights to different levels of features through the ASFF module to increase the importance of key levels and also using the UNet structured network combined with an attention mechanism to predict the depth information, which also extracts the key area information. It aims to improve the performance and accuracy of the TransMVSNet algorithm’s 3D reconstruction of UAV remote sensing images. In this work, we have performed comparative experiments and quantitative evaluation with other algorithms on the DTU dataset as well as on a large UAV remote sensing image dataset. After a large number of experimental studies, it is shown that our improved TransMVSNet algorithm has better performance and robustness, providing a valuable reference for research and application in the field of 3D reconstruction of UAV remote sensing images.

## 1. Introduction

In recent years, multi-view stereo (MVS) has become a research hotspot in the field of computer vision, with high potential value in computer-aided design [1], virtual reality [2], augmented reality [3], and robot navigation [4]. Prior to deep learning, conventional multi-view stereo vision involved first calibrating the camera [5], i.e., calculating the camera’s image coordinate system in relation to the world coordinate system. Information from multiple 2D images was then used to reconstruct 3D information. Although great achievements have been made in the reconstruction of Lambertian surfaces [6], they are still affected by factors such as illumination variations or low texture leading to poor reconstruction results.

The three-dimensional reconstruction of remote sensing scenes by unmanned aerial vehicles (UAVs) is receiving increasing attention. However, there are still some challenges in the 3D reconstruction process, including the accuracy and efficiency of reconstruction, especially in complex and large-scale scenes. Therefore, the problem to be addressed in this study is the accuracy and efficiency of the 3D reconstruction of remotely sensed scenes using UAVs, especially in complex and large-scale scenes. The specific objective of this research is to improve the existing TransMVSNet 3D reconstruction algorithm with the aim of providing more accurate and detailed 3D models for various scenes. The importance of this work is that it has the potential to improve the adaptability of multi-view vision in the field of unmanned remote sensing and contribute to the development of better algorithms.

### Related Work and Contribution

Among the traditional 3D reconstruction algorithms, the most representative of sparse reconstruction is the Structure from Motion (SFM) [7] algorithm, which takes a set of images as input and generates two pieces of information: the camera parameters of each image and a set of 3D points visible in the image, which are usually encoded as trajectories. A trajectory is defined as a list of the 3D coordinates of the reconstructed 3D points and the corresponding 2D coordinates in a subset of the input images. Dense reconstruction [8] is the process of finding points in space with photometric consistency [9] to stereo match the scene when the camera parameters are known. On these bases, OpenMVS [10] is a more classical multi-view stereo (MVS) open-source library that integrates the complete technical solution for 3D reconstruction, which includes camera modeling, multi-view stereo, dense reconstruction, surface reconstruction [11], point cloud fusion [12], and texture mapping [13].

In the era of deep learning, deep learning-based stereo vision uses deep CNNs to extract the depth map for each view, and the 3D model is finally obtained through multi-view fusion, which can effectively improve the accuracy of 3D reconstruction. In particular, MVSNet [14] proposed by Yao Yao et al. is the originator of multi-view stereo vision. In this approach, the algorithm mainly extracts the depth map for each view, and central to this is that each pixel of the reference image is searched along the antipodal line in all the projection-transformed source images to find the best depth with the lowest matching cost. Consequently, a depth map with high resolution and accuracy can be obtained. Then, in 2019, Prof. Long Quan’s team at the Hong Kong University of Science and Technology improved MVSNet and proposed RMVSNet [15], which replaced 3D convolution with the GRU temporal network to reduce the model size, and then the loss was changed to cross-entropy loss for multi-classification.

In 2019, Chen et al. proposed PointMVSNet [16] based on MVSNet, which is an algorithm that predicts the depth information and then forms a 3D point cloud using the image. It then uses the algorithm of the 3D point cloud to optimize the regression of depth. Subsequently, in 2020, Hongwei Yi et al. proposed PVA-MVSNet [17], which is an algorithm that uses the attention mechanism to adaptively learn weights, such as the weights of different viewpoints. X. Gu et al. also proposed the Cascade-MVSNet [18] algorithm, which utilizes the strategy of chained costume construction to estimate the coarser depth values first and then further reduces the depth estimation range to improve the depth estimation accuracy, achieving higher resolution and higher accuracy depth maps with less GPU consumption. After dense reconstruction, the results of Cascade-MVSNet are also more complete than those of the previously mentioned methods. Meanwhile, in 2020, Jiayu Yang et al. proposed an unsupervised neural network, CVP-MVSNet [19]. This is a costume-based, small, and computationally efficient MVS deep inference network. The costume pyramid is constructed in a coarse-to-fine manner based on a detailed analysis of the relationship between the depth residual search range and image resolution; the framework can use less memory to process higher resolution images. It is six times faster than PointMVSNet.

More recently, Ding, Yikang et al. proposed the TransMVSNet [20] algorithm, which is the first attempt to use Transformer for MVS tasks. The authors utilize MVS due to its suitability for feature matching tasks and propose a powerful Feature Matching Transformer (FMT) [21] to leverage intra- (self-) and inter- (cross-)attention [22] to aggregate long-range context information within and across images. In this paper, to facilitate a better adaptation of the FMT, we leverage an Adaptive Receptive Field (ARF) [23] module to ensure smooth transfer in terms of the scope of features and bridge different stages with a feature pathway to transfer transformed features and gradients across different scales.

The algorithms mentioned above provide outstanding contributions to the field of multi-view stereo (MVS). Moreover, the application of 3D reconstruction technology in the field of UAV remote sensing [24] has been expanding, providing richer information and data for topographic surveying [25], urban planning [26], environmental monitoring [27], and other fields. By carrying photographic equipment for aerial photography using unmanned aircraft and processing and analyzing aerial images with 3D reconstruction algorithms, high-precision and high-resolution 3D maps [28] and models can be constructed, providing data support for other geographic information systems [29] and geoscientific research [30]. Compared with traditional multi-view stereo (MVS) vision datasets, the scenes in UAV remote sensing images are more complex, the image resolution is larger, and the impact of weather and other factors may lead to lower texture clarity in the image. The TransMVSNet network adopts the Transformer module, which is more suitable for this kind of scene, so our work is based on this.

In this work, we improve the TransMVSNet network, which uses a feature extraction module (FPN) [31] as its feature extraction network. In this paper, we use an asymptotic feature pyramid network (AFPN) [32] to support the direct interaction of non-adjacent layers. AFPN is initiated by fusing two neighboring low-level features and progressively incorporating high-level features into the fusion process. In this way, large semantic gaps between non-adjacent levels can be avoided. Considering the possibility of multi-target information conflicts during the feature fusion process at each spatial location, adaptive spatial fusion operations are further utilized to mitigate these inconsistencies. We use a network module based on the UNet structure [33] combined with an attention mechanism to improve the original network module in the construction of the cost volume, which can improve the quality and the accuracy and reliability of the generated depth map.

To summarize, our contributions are as follows:

(1) We improve the TransMVSNet neural network to estimate the depth map of UAV remote sensing images and solve the information conflict in the depth extraction process that results in unreliable depth maps.
(2) We adopt an asymptotic feature pyramid network (AFPN) that progressively integrates low-level, high-level, and top-level features in the bottom–up feature extraction process of the backbone network [34]. Meanwhile, different spatial weights are assigned to the features of different levels by ASFF [35], which enhances the importance of key levels and mitigates the effect of contradictory information from different targets.
(3) We use a UNet neural network to predict the depth while incorporating an attention mechanism to extract critical region information by adding weights.

## 2. Proposed Methods

### 2.1. Feature Extraction

TransMVSNet adopts the FPN feature pyramid structure, comprising a top–down path to achieve the fusion of features of different levels and a bottom–up path to make up for the lack of low-level feature details in high-level features. However, detailed information from low-level features may be lost or degraded during propagation and interaction. This leads to suboptimal extraction of detailed features from the original view.

**Figure 1:**
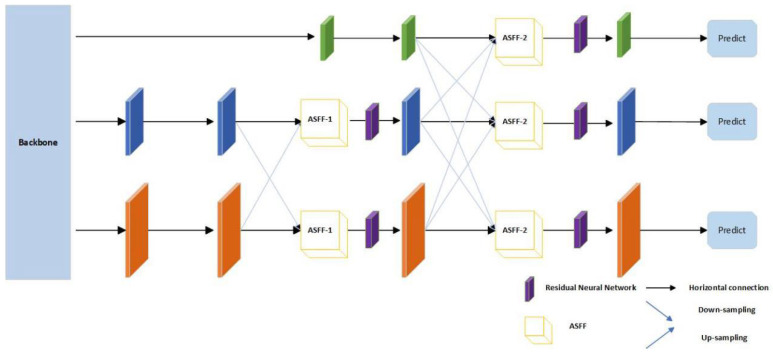
The architecture of the proposed asymptotic feature pyramid network (AFPN). AFPN is initiated by fusing two neighboring low-level features and progressively incorporating high-level features into the fusion process.

In our work, we introduce a novel feature extraction pyramid (AFPN) with the structure shown in Figure 1. The bottom–up feature extraction process in the backbone begins by fusing two low-level features of different resolutions. In the later stages of feature extraction, the high-level features are incorporated into the fusion process to finally fuse the top-level features of the backbone. This type of fusion avoids large semantic gaps between non-adjacent levels.

In this process, low-level features are fused with semantic information from high-level features, and high-level features are fused with detailed information from low-level features. Due to this direct interaction, information loss or degradation in multi-level transmission can be avoided. This solves the above-mentioned limitations.

Based on the overall network architecture of the TransMVSNet network, the AFPN structure extracts three multi-scale depth image features with resolutions ranging from coarse to fine. In the overall feature extraction structure, the features are first coarsely extracted using a sequence of three simple network layers, the combination of which is a Conv2d network layer, a batchnormed normalization layer, and a Sigmoid Linear Unit (SiLU) [36] activation function. The SiLU activation function is smoother as it approaches 0, which can make the network output range between 0 and 1, which is more applicable in this structure. It is defined as follows:

(1) $$silu=x*sigmoid\left(x\right)=\frac{x}{1+e^{-x}}$$

Next, we input the features into the AFPN after changing the number of feature channels. In the AFPN structure, we used a 2 × 2 convolution with stride of 2 for 2-fold downsampling and a 4 × 4 convolution with stride of 4 for 4-fold downsampling; we used a similar structure for upsampling. This adapts the Adaptively Spatial Feature Fusion (ASFF) module. This is the most important aspect of our AFPN network, as it solves the problem of inconsistency within the feature pyramid by learning the links between different feature maps.

Next, we employ the ASFF [37] neural network model, which aims to improve the effectiveness of feature fusion and hence the accuracy of the deep costumers. The ASFF network model introduces an adaptive feature fusion module for fusing multiple feature maps at different levels. After the AFPN backbone network extracts the feature maps at different levels, it uses the adaptive attention mechanism to weight the fusion of these feature maps. In order to better retain the important feature information, compared to the feature extraction network without the ASFF network model, the multi-scale information can be better extracted after the addition, so that better results can be achieved in the next deep costal body construction.

In the ASFF module, for a certain level of features, the other levels are first adjusted to the same resolution and simply integrated, and then they are trained to find the best fusion. In Figure 1, we assume that before entering the ASFF-2 module, the feature levels are x1, x2, and x3. By multiplying the weight parameters α, β, and Ƴ for the features from different layers and summing them up, the new fused features are obtained as shown in the following equation:

(2) $$y_{ij}^{l}=\alpha_{ij}^{l}\cdot x_{ij}^{1\to l}+\beta_{ij}^{l}\cdot x_{ij}^{2\to l}+\gamma_{ij}^{l}\cdot x_{ij}^{3\to l}$$

As can be seen from Equation (2), the ASFF module uses summation, which requires that the three level layers output the same-sized features and the same number of channels. Therefore, it is necessary to perform upsampling or downsampling and adjust the number of channels for the features of different layers, as described above.

The weight parameters α, β, and Ƴ are then obtained by adjusting the feature maps of the different layers after a 1 × 1 convolution, followed by softmax after concat so that they are all in the range of [0, 1] and sum to one:

(3) $$\alpha_{\mathrm{ij}}^{l}=\frac{e^{\lambda_{\alpha_{ij}}^{l}}}{e^{\lambda_{\alpha_{ij}}^{l}}+e^{\lambda_{\beta_{ij}}^{l}}+e^{\lambda_{\gamma_{ij}}^{l}}}$$

The λ in Equation (3) represents the coefficients of the softmax function, meaning that the weight parameter can be used to adjust the size of the contribution of each feature map during the feature fusion.

Following the ASFF module, it is possible to enhance the importance of the critical level feature map and mitigate the impact of conflicting information from different targets. After feature fusion, we input each feature into the residual unit to continue learning features. As shown in Figure 1, we use a total of five residual units; each residual unit is similar to ResNet, including two 3 × 3 convolution modules, two batchnormed normalization layers, and a SiLU activation function. The residual unit maps the input and output to Equation (4), which allows for better feature learning and also optimizes the training of the neural network.

(4) $$y=H\left(x,w_{h}\right)+x$$

In Equation (4), H(x, w_h_) represents a residual unit, and x is the direct mapping component.

Through the above work, we can extract more detailed image features through the AFPN feature pyramid. After fusion with the Transformer model, its powerful self-attention mechanism and positional coding method can be used to extract and aggregate image features more accurately, laying a solid foundation for subsequent deep extraction.

### 2.2. Refinement of the Depth Forecast

The initial cost volume computed from the image feature maps is likely to contain noise, mainly due to problems related to the presence of non-Lambertian surfaces or line-of-sight occlusion.

Therefore, in order to predict the depth map, it is necessary to smooth the initial cost volume, optimize it, and refine the probability volume. In the original TransMVSNet network, a multi-scale 3D-CNN network is used for cost volume regularization. This network is similar to the 3D version of UNet, using an encoding–decoding architectural approach for neighborhood information aggregation over a large range of sensory fields at a relatively small cost volume.

Due to the fact that the global information is weighted on the same scale during the calculation process, it is not possible to suppress the information in regions of the image that are not related to the target object. The results predicted from each angle result in large errors at the edges and are smoother; thus, they fail to reflect the depth difference and make the reconstructed target hierarchy indistinct.

**Figure 2:**
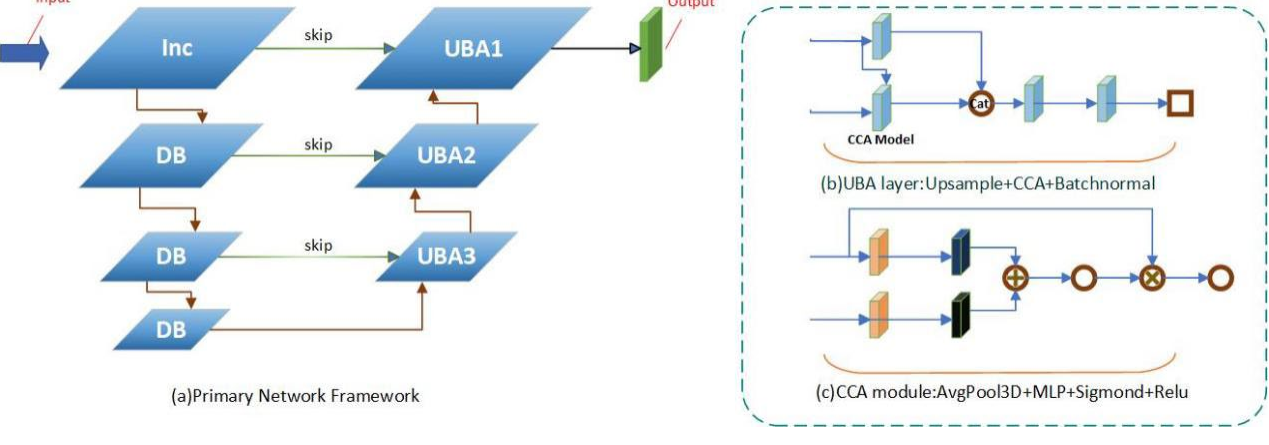
Cost volume regularization network: (**a**) the overall network, (**b**) the UBA layer, and (**c**) the CCA module in the UBA layer.

In order to solve this problem, the attention mechanism is added to the existing UNet structure in this work. The schematic is shown in Figure 2.

First, we applied the cross-attention module CCA [38]; the current level feature map and the previous level feature map are used as the first inputs into the CCA.

These two feature maps first travel to the AdaptiveAvgPool3d network layer, whose main role is to perform an adaptive average pooling operation on the input 3D data. Suppose the output data are N × h × w × d, where h is the height, w is the width, and d is the depth of the output data. The operation of the AdaptiveAvgPool3d module can be expressed as follows:

(5) $$output[i,j,k]=1/\left(h\times w\times d\right)*sum\left(input\left[p,q,r\right]\right)$$

In Equation (5), the parameters satisfy the following conditions:

(6) i=h/H×p;j=w/W×q;k=d/D×r

**Figure 3:**
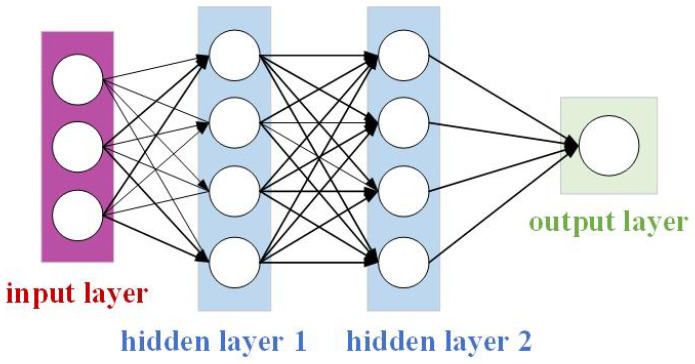
Fully connected (FC) network structure.

After the AdaptiveAvgPool3d module adaptively tunes the features, they pass into the fully connected (FC) [39] network structure, which is shown in Figure 3.

In the fully connected network, the input layer has n neurons, the hidden layer has m neurons, and the output layer has k neurons. The input of the input layer is denoted by x, the output of the hidden layer by h, the output of the output layer by y, the weight matrix by W, and the bias vector by b. Then, the computation process of the fully connected layer can be expressed as follows:

(7) $$h=f\left(W_{1}x+b_{1}\right)$$

(8) $$y=g\left(W_{2}h+b_{2}\right)$$

The computation of the hidden layer is shown in Equation (7), and the computation of the output layer is shown in Equation (8), F and g. The two formulas contain the activation functions; the dimensions of weight matrix W1 are (m, n), and the dimensions of W2 are (k, m); the dimensions of bias vector b1 are (m, 1), and the dimensions of b2 are (k, 1). So, by continuously iteratively adjusting the weights and biases, fully connected neural networks can learn complex nonlinear relationships between inputs and outputs.

Here, we assume that the input two feature maps are X and Y, and their dimensions are N × C and N × D, where N denotes the size of the feature map, and C and D denote the number of channels of the feature map, respectively. First, we compute the attentional weight matrices of X and Y, denoted as AX and AY, respectively. These weights are obtained by calculating the correlation between the two feature maps. Specifically, we can calculate the attentional weights between X and Y using the following formula:

(9) AX=softmax⁡(XW_qY^T)

(10) AY=softmax⁡(YW_qX^T)

In Equations (9) and (10), W_q is the query weight matrix. In this way, the CCA module can obtain the spatial location correlation on the feature map, and finally the spatial location correlation weight scale can be obtained via the Sigmoid activation operation.

The overall network structure is similar to that of the UNet network, where features are first downsampled in 3D and then upsampled, and attention is computed with the features in the downsampling process to obtain the feature relevance weight scale. This suppresses irrelevant regional features in the image by changing the weights while highlighting the significant features of the feature regions, especially the depth information of the edge mutation regions. This makes the predicted depth results hierarchical and more accurate.

## 3. Experimental Results

The DTU dataset [40] and a self-built UAV ground loop shot and push–sweep dataset were used to evaluate and validate our approach.

The DTU was captured using a fixed camera track in a well-controlled laboratory environment and contains 128 scenes in 49 views under seven different lighting conditions. Referring to the MVSNet network setup, we categorized the dataset into 79 training scenes, 18 test scenes, and 22 evaluation scenes.

We trained the original TransMVSNet network with the DTU dataset as well as our improved TransMVSNet network, and we used both the Pytorch training framework and cuda version 11.1 to train the network. In the training phase, we set the number of input images to N = 5 and the image resolution to 512 × 640. For coarse-to-fine regularization, the depth hypotheses were sampled from 425 mm to 935 mm; the number of depth hypotheses for each stage was 48, 32, and 8, respectively. The corresponding depth intervals were attenuated from the coarsest stage to the finest stage by 0.25 and 0.5, respectively.

**Figure 4:**
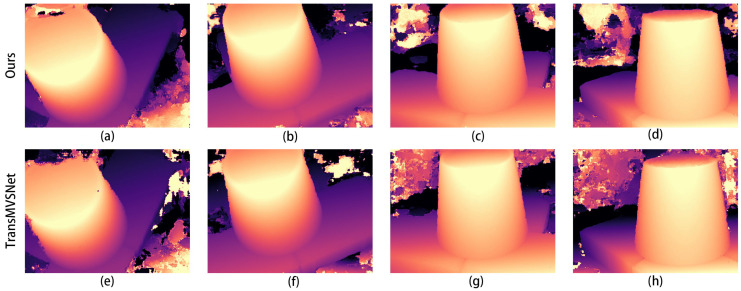
Comparison of depth prediction results for Scan1, where (**a**–**d**) are the results of our algorithm and (**e**–**h**) are the results of the original algorithm.

Then, we tested the optimal weights of the training results separately on the test dataset. A comparison of the depth maps of the images in the Scan1 scene is shown in Figure 4.

In the images in Figure 4a,e, it can be seen that the improved algorithm extracts more complete edges of the depth image and has less clutter in the background. The other results from b to g also show a more distinct and complete hierarchy in places in the mutated region at the edge of the depth map.

**Figure 5:**
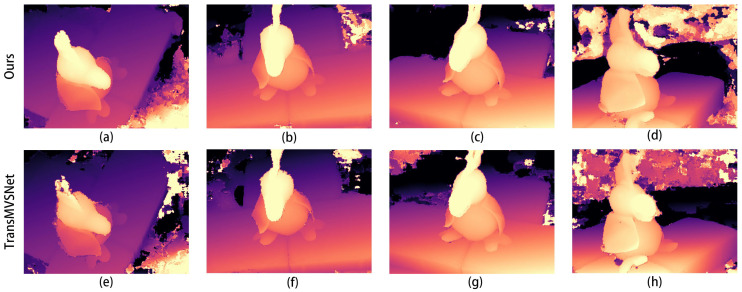
Comparison of depth prediction results for Scan4, where (**a**–**d**) are the results of our algorithm and (**e**–**h**) are the results of the original algorithm.

**Figure 6:**
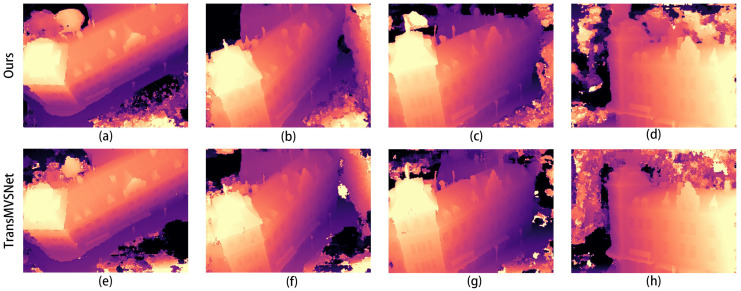
Comparison of depth prediction results for Scan9, where (**a**–**d**) are the results of our algorithm and (**e**–**h**) are the results of the original algorithm.

**Figure 7:**
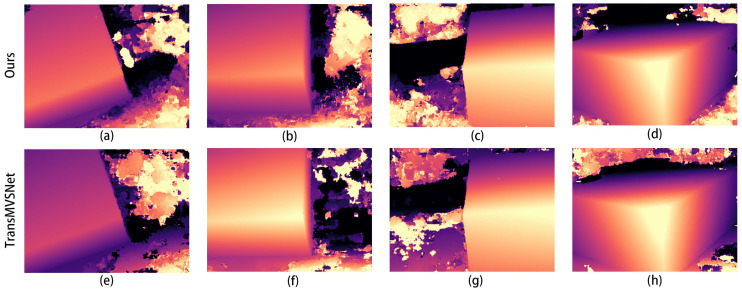
Comparison of depth prediction results for Scan10, where (**a**–**d**) are the results of our algorithm and (**e**–**h**) are the results of the original algorithm.

The comparison graphs of the Scan4, Scan9, and Scan10 scenes in the DTU dataset are shown in Figure 5, Figure 6 and Figure 7.

In Figure 5, Figure 6 and Figure 7, (a) to (d) are the depth prediction results of our improved algorithm, and (e) to (h) are the depth prediction results of the original algorithm. From the comparison results, the improved algorithm can be seen to outperform the original algorithm in terms of the accuracy, completeness, and detail of depth prediction. This is mainly due to the optimization of the improved algorithm in terms of feature extraction and depth prediction.

In the feature extraction stage, the AFPN structure can adaptively fuse features of different scales, which enhances the feature representation. In the depth prediction stage, large-scale depth variation and detail information can be better handled by introducing a coarse-to-fine regularization strategy.

**Figure 8:**
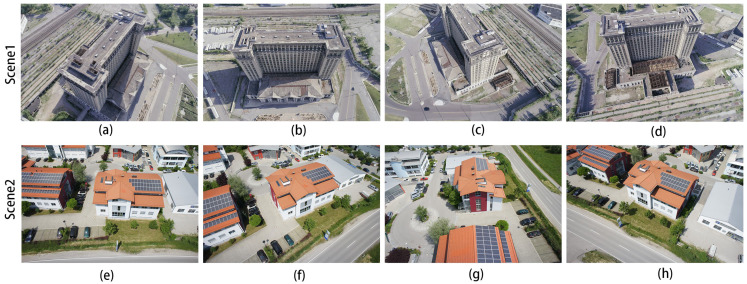
(**a**–**h**) are overhead drone images of buildings from the drone mapping dataset Pix4D. Scene 1 is an unfinished building and Scene 2 is a residential home in Chicago, IL, USA.

To evaluate the application of our model in the field of UAV remote sensing, we also performed the corresponding test in the main scene reconstruction of buildings, as shown in Figure 8. Here, the image of Scene 1 was obtained from a public dataset provided by the well-known UAV photogrammetry company PIX4D [41]. The camera model was DJI FC6310, the world coordinates were selected as WGS84/UTM zone 17N, and the image resolution was 5472 × 3478. The image for Scene 2 was captured with an AV-900 model drone with a Sony NEX-5T camera model; the world coordinate system was also selected as WGS84/UTM zone 17N, the average ground sampling distance (GSD) was 1.66 cm/0.65 in, and the image resolution was 4912 × 3264.

For both scene datasets, we extracted the internal and external camera parameters separately. After adjusting the format of the data to that of DTU, they were fed to both our improved algorithm as well as the original algorithm for depth extraction.

**Figure 9:**
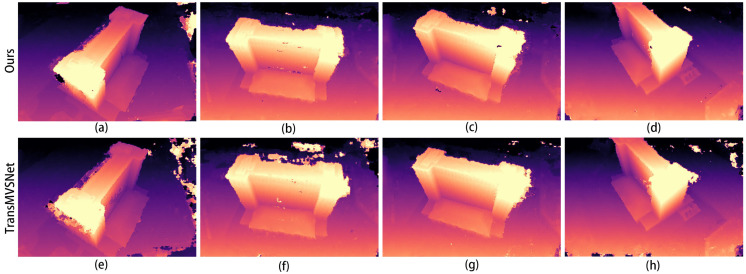
Depth map of the first scene in Figure 8: (**a**–**d**) are depth prediction images of our improved algorithm; (**e**–**h**) are depth prediction images of the original algorithm.

**Figure 10:**
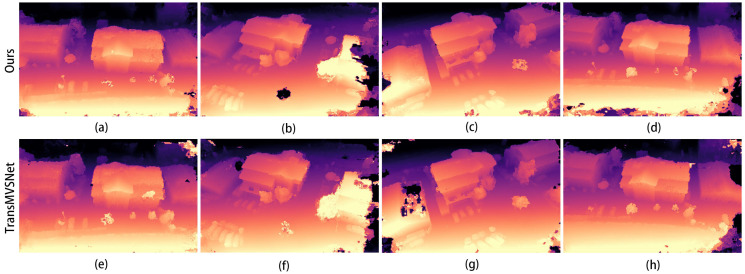
Depth map of the second scene in Figure 8: (**a**–**d**) are depth prediction images of our improved algorithm; (**e**–**h**) are depth prediction images of the original algorithm.

The corresponding depth map results for the two scenarios in Figure 8 are shown in Figure 9 and Figure 10. In both figures, (a–d) show the depth prediction images of the improved algorithm and (e–h) show the depth prediction images of the original algorithm.

As can be seen from Figure 9a,e, the edges of the building in Figure 9e are not smooth and appear jagged. After employing our improved algorithm, the edges of the building are smoother, and the depth information of the mutated region is more accurate.

**Figure 11:**
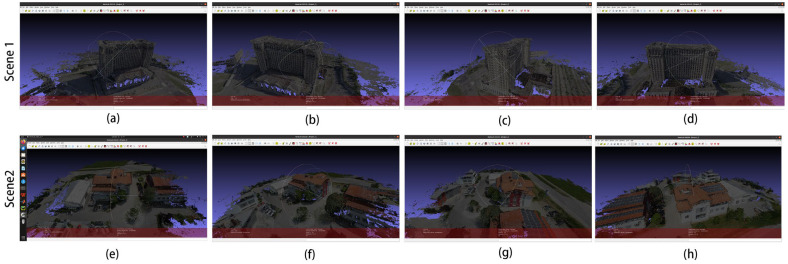
The 3D reconstruction results of the improved algorithm: (**a**–**d**) for Scene 1 and (**e**–**h**) for Scene 2 in Figure 8.

These two scenes belong to the UAV ring shot dataset. The camera is positioned at a fixed attitude angle around the scene, similar to the DTU dataset, and the reconstruction of such scenes is also good. The reconstruction results are shown in Figure 11, in which (a–c) are the reconstruction results of Scene 1, and (d–f) are the reconstruction results of Scene 2.

The 3D models in Figure 11a–d are the 3D reconstruction results of Scene 1 in Figure 8, and (e–h) are the 3D reconstruction results of Scene 2 in Figure 8. This 3D model was created with MeshLab v2022.02 [42] software, and we used a part of the model under different angles for illustration; the following 3D model also uses the same method.

**Figure 12:**
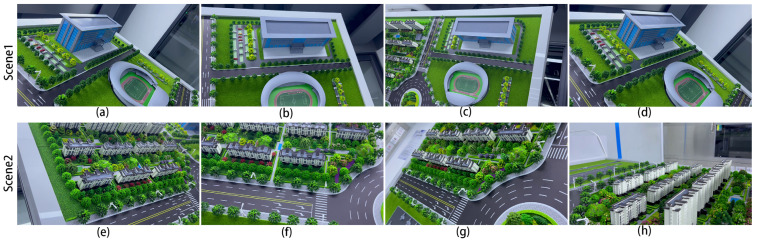
Self-constructed wrap-around robotic arm overhead shooting scene dataset: (**a**–**d**) are Scene 1, which mainly includes schools and stadiums; (**e**–**h**) are Scene 2, which mainly includes residential areas.

We also built two of our own datasets to validate our algorithms, as shown in Figure 12. We prepared a sandbox demonstration system for ground targets, and we used a robotic arm to drive the camera around to capture the image. The camera’s focal length is 26 mm, the aperture size is 1.5, and the resolution of the captured image is 1080 × 1920.

**Figure 13:**
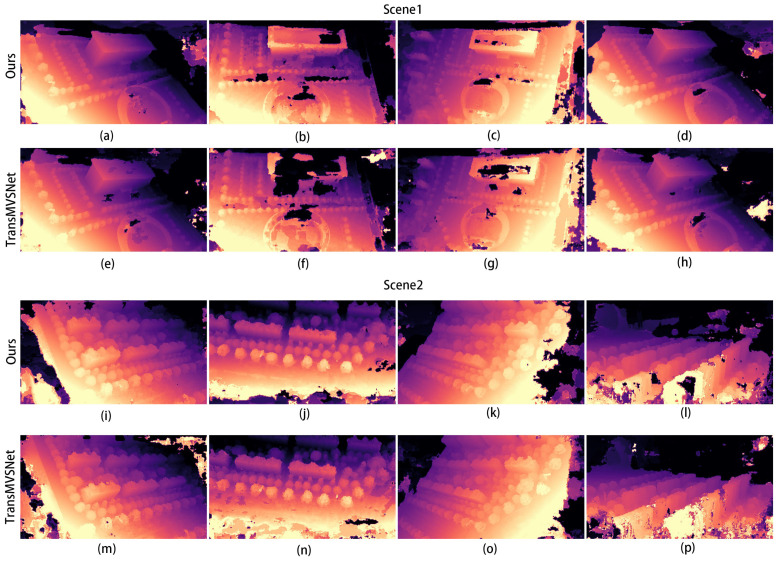
(**a**–**p**) contain the comparison of the depth maps of the two self-built scenario datasets: Scene 1 is the result of Figure 13, and Scene 2 is the result of Figure 14.

The results for the two scenes in Figure 12 are shown in Figure 13, and it can be seen clearly that the edges of the building are clearer and there is less depth loss in the improved algorithm between Figure 13b and Figure 13e. From Figure 13b,e, it can be clearly seen that the edges of the building are clearer, and less depth is missing in the results of the improved algorithm. Similarly, the comparison between Figure 13i and Figure 13l shows the same effect. Figure 16 shows the 3D reconstruction model produced using our improved algorithm.

**Figure 15:**
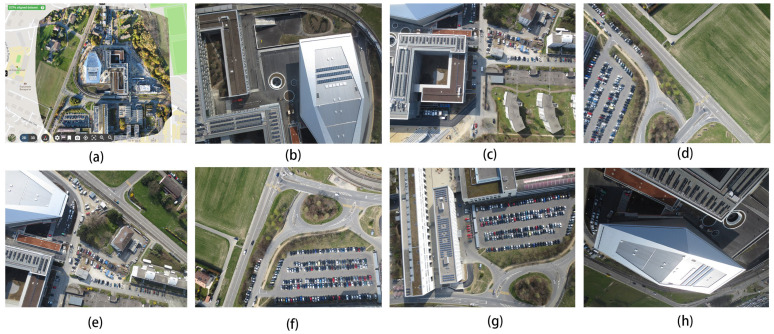
(**a**–**h**) are iconic site in Lausanne, showcasing the city’s beauty and history. It is the capital of the canton of Vaud, Switzerland, from the unmanned remote sensing dataset Pix4D.

Next, we acquired a dataset of push–sweep photography captured with a drone and a publicly available dataset from the photogrammetric company Pix4d, whose camera model is the Canon IXUS 127 HS, and selected the world coordinate system as WGS84/UTM zone 32N. Because the ground sampling resolution is small and the scene as a whole is very large, we selected a few images to be displayed in Figure 15.

**Figure 16:**
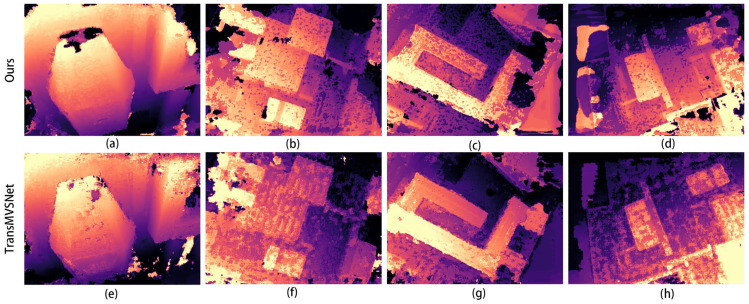
Depth prediction image of the scene in Figure 15, comparing our improved algorithm with TransMVSNet, where (**a**–**d**) are the results of our algorithm and (**e**–**h**) are the results of the TransMVSNet.

**Figure 17:**
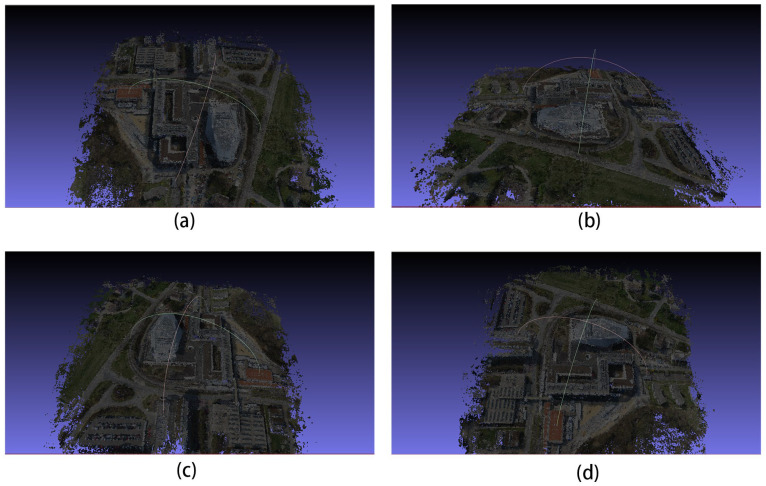
(**a**–**d**) are the three-dimensional reconstructed model view of the scene in Figure 15.

The depth maps in Figure 16 contain the more iconic buildings in this scene. From the comparison of Figure 16c,g, for example, the edges of the buildings in Figure 17c are smoother and the layers are clearer, while the depth of the buildings in Figure 16g is more pronounced, which can also show that our improved algorithm is very effective in improving the results. Figure 17 shows the 3D reconstruction model created using our improved algorithm.

**Figure 18:**
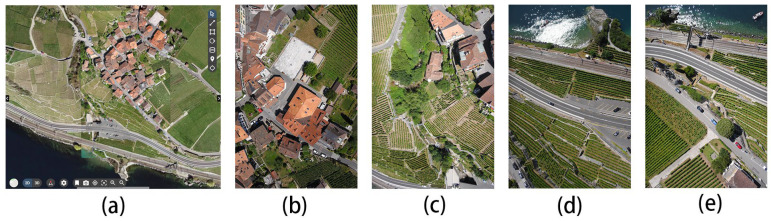
(**a**–**e**) show coastal mountain village in the capital of the canton of Vaud, district of Lausanne, Switzerland, photographed on Pix4D.

The scenes in Figure 15 are mainly urban scenes. We then found an image of a scene with a mountain village and a natural landscape, located in the capital of the canton of Vaud, district of Lausanne, Swiss Confederation. This dataset was obtained with the camera model senseFly S.O.D.A, and the coordinate system used is CH1903+/LV95. The full view of the scene in the dataset is shown in Figure 18a; b–e are the parts of the images that were fed into the neural network, which have a resolution of 3648 × 5472.

**Figure 19:**
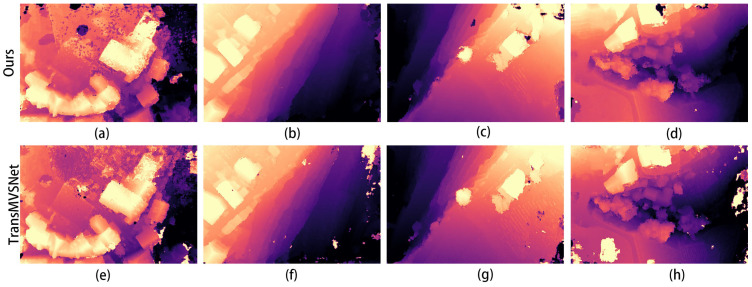
Depth map of the scene in Figure 18: (**a**–**d**) are depth prediction images of our improved algorithm; (**e**–**h**) are depth prediction images of the original algorithm.

**Figure 20:**
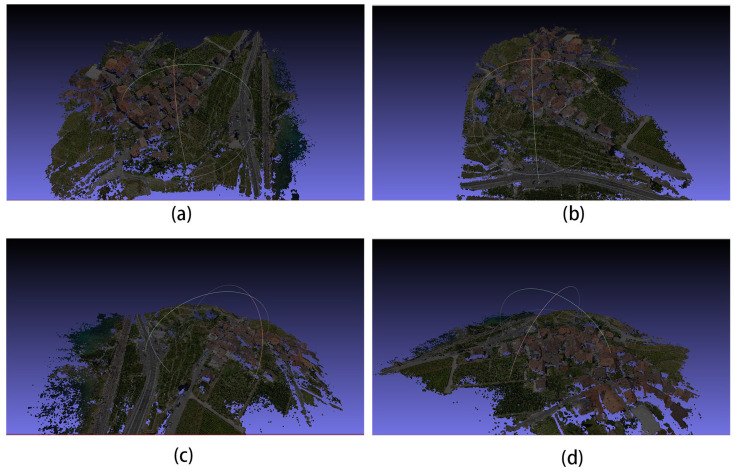
(**a**–**d**) are the three-dimensional reconstruction model view of the scene in Figure 18.

Figure 19 shows the computed depth image and the results of the 3D reconstruction are shown in Figure 20 for each of the four views of the model.

## 4. Discussion

### 4.1. Comparison on the DTU Dataset

The main difference between our algorithm and TransMVSNet is the use of the multi-scale feature extraction network, which can provide multi-scale features for the Transformer to better compute inter-image and intra-image attention and, at the same time, change the global information weights when predicting the depth information. This can effectively inhibit the irrelevant regions in the image with the target, making the prediction results more accurate. We also compare it with the MVS algorithm mentioned in the Introduction section.

**Figure 21:**
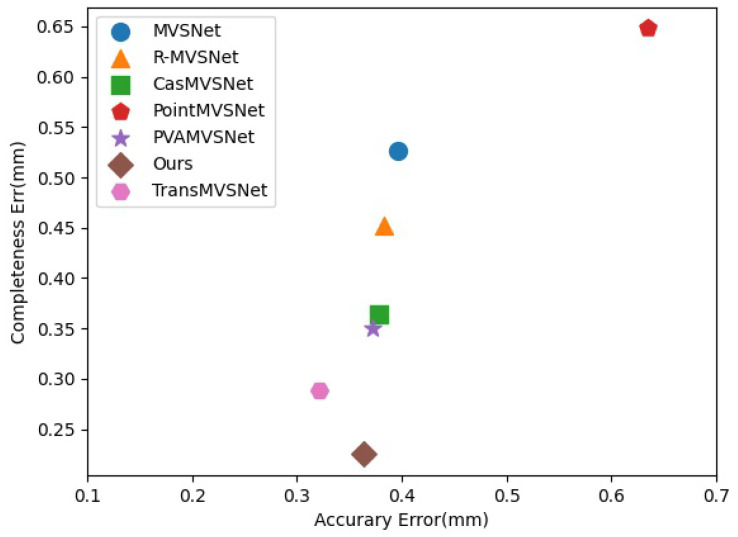
Comparison with state-of-the-art deep learning-based MVS methods on DTU dataset (lower is better).

In the DTU dataset, there are two metrics to evaluate the accuracy of the point cloud: Acc and Comp. The Acc metric is used to find the points in the reconstructed point cloud according to the points in the reconstructed point cloud and evaluate the accuracy of the point cloud by the distance between the points in the reconstructed point cloud and the points in the reconstructed point cloud. And the Comp metric is used to find out whether there are any corresponding points in the reconstructed point cloud based on the points in the true value of the point cloud and to evaluate the integrity of the point cloud by the number of corresponding points. The lower the values of the metrics, the better, as this means that the distance between the reconstructed resultant point cloud and the true value of the point cloud is smaller and thus means that the recovered point cloud is more accurate. The comparison of our algorithm with several other algorithms is shown in Figure 21.

In Table 1, the comparison of the accuracy of these algorithms on the DTU dataset is shown. From the data in the table, it can be seen that our algorithm is ranked second in Acc and first in Comp. The overall value is the average of the first two metrics; so, based on this comparison, our algorithm performs better than the other algorithms on the DTU dataset.

In Table 2, it can be seen that for the same input size, our algorithm and TransMVSNet have reduced inference speed while requiring more GPU memory for training. This is what we need to optimize in subsequent neural networks.

### 4.2. Comparison on UAV Remote Sensing Datasets

The main reasons for why we chose to improve TransMVSNet are the shortcomings related to the weak texture, repetitive texture, and non-Lambertian surfaces of UAV remote sensing images, which have a large impact on the results in the multi-view stereo (MVS) process. Moreover, the process of multi-view stereo (MVS) is a one-to-many feature matching process, which uses convolution to take local features. The localization of convolutional features hinders the perception of global information, which is crucial for robust depth estimation in regions with the above-mentioned shortcomings. TransMVSNet combines Transformer with multi-view stereo (MVS), and our work improves the feature extraction and depth prediction part of Transformer by considering the global feature information and discarding the feature information in the useless region, which makes the depth prediction result more accurate.

Meanwhile, our algorithms are very sensitive to the settings of hyperparameters, which include numdepth, depth hypotheses, and depth interval. In the DTU dataset, these three hyperparameters are set the same as in TransMVSNet to make it convenient to compare the results. In the dataset of remote sensing images from UAVs, we needed to adjust the hyperparameters to ensure that it achieved the best depth prediction. The numdepth was set to 192 in almost all MVS series algorithms. Therefore, we also used 192, followed by the depth interval hyperparameter (4, 1, 0.5), and finally set the depth hypotheses; this hyperparameter represents the number of plane scanning depth hypotheses for each stage and is generally set to three stages. After the first stage, with the maximum number of hypotheses, it can subsequently become smaller because the larger the setting, the smaller the depth interval. The initial stage requires strong supervision to be able to output more details and later becomes smaller to make the details smoother. Therefore, this is also a factor that can determine whether the depth prediction is accurate.

**Figure 22:**
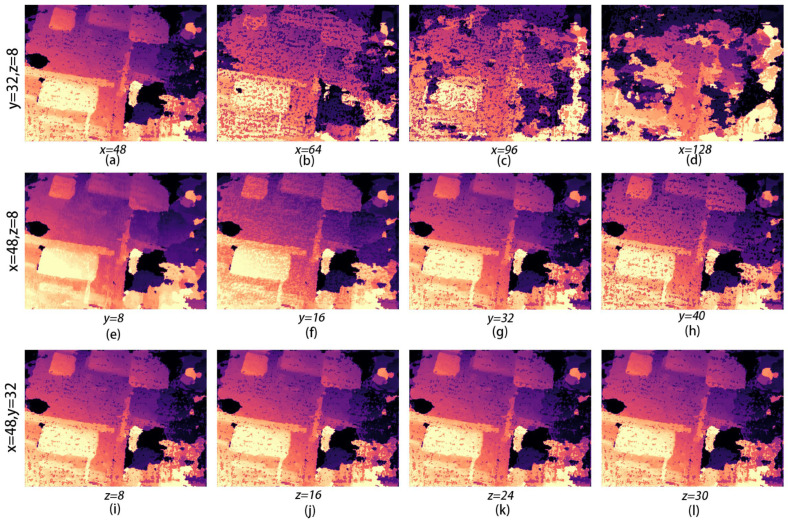
Quantitative experiments: (**a**–**d**) are the first to quantify the number of scans in three planes, (**e**–**h**) are the second to quantify the number of scans in three planes, and (**i**–**l**) are the third to quantify the number of scans in three planes.

To set the hyperparameter depth hypotheses, we carried out quantitative experiments. First, we set the number of small two-plane scans in the three phases to a fixed value (32, 8) and changed the maximum number of plane scans x, which was set to 48, 64, 96, and 128. Then, we changed the number of second-plane scans y, which was set to 8, 16, 32, and 40, and set the other two phases to a fixed value (48, 8). Finally, we changed the number of third-plane scans in the three phases, which was set to 8, 16, 24, and 30, and set the other two phases to fixed values (48, 32). The results are shown in Figure 22.

The results of the quantitative experiments are shown in Figure 22. When y = 32 and z = 8, the first phase of the plane scanning depth x is 48. Here, the depth prediction result is the best; its depth error is small, and the edge of the object is smoother. Then, we set x = 48 and z = 8, and the best results are obtained when the second phase of the scanning depth y is 32, as shown in Figure 22g. When y = 8, the depth is not very obvious, but it is slightly better than at y = 16, and there is still a large part of the image missing. Finally, when we set x = 48 and y = 32, the best result is obtained when the third phase of the plane scanning depth z is 8. In the four images in Figure 22i–l, it does not look like the difference is very large because the third phase of the plane scanning depth is small, resulting in large depth intervals and less detailed information. So, although the difference is not very large, based on the smoothness of the object edges, z = 8 can be judged to be the best parameter setting. Therefore, the super parameter depth hypothesis is set to 48, 32, 8.

### 4.3. Constraints or Challenges

When we improved the feature extraction network model, first of all, the complexity of the AFPN network model was much larger than that of the FPN, so the parameters of each network layer needed to be constantly debugged to obtain the optimal collocation. Meanwhile, considering that after extracting multi-scale features, we need to assign weights to the key features to achieve the optimal feature extraction, we adopted the ASFF network model.

During the algorithmic research, after replacing the FPN feature extraction network model using the AFPN combined with the ASFF feature extraction network model, considering updating the FMT feature matching module of the original network, after checking the related literature, we found that the local feature transform LoFTR [43] network model can satisfy our needs, and this network model also uses the self- and cross-attention layer. This network model also uses a Transformer with self- and cross-attention layers to handle the dense local features extracted from the backbone network. After our modification, we found that there is not much difference in the idea and algorithm structure with the original network, so there is no big improvement, so we discarded this improvement.

In the final stage of designing the costume regularized network model, we continuously optimize the network structure and hyperparameter combinations and finally determine the best network design scheme to incorporate the cross-attention module CCA into the UNet network model, and at the same time, in order to optimize the training process of the model, we use two NVIDIA 3090 graphics cards for distributed training to improve the training efficiency.

All of the above are challenges and limitations that we encountered during the design and research phase of the algorithm, and we eventually overcame them to ensure the stability and reliability of the research model.

## 5. Conclusions

In this paper, we improve the TransMVSNet network by first extracting the cascade features using the AFPN feature extraction network and then improving the regularized costumed body network by incorporating an attention mechanism to make the overall network suitable for the 3D reconstruction of images captured by UAVs in the field of remote sensing. We combined the characteristics of the remote sensing images taken by UAVs, i.e., the image features are richer, the field of view is wider, and the ground buildings occupy fewer pixels in the image; therefore, our improved network was better able to extract the image features, highlight the information of the feature area, and make the edges of the objects more hierarchical.

In previous 3D reconstruction work, researchers have focused mainly on close-up object reconstruction and urban outdoor scene reconstruction. Three-dimensional reconstruction in the field of remote sensing is also a research focus. Our reconstruction method can completely restore the whole image of an area, which can provide valuable help for remote sensing measurements and other subsequent work.

Our algorithm also has limitations in the field of UAV remote sensing 3D reconstruction. First of all, the input image sequence must be continuous and comprehensive; if there are many missing images, it will lead to missing depth prediction, resulting in an incomplete reconstructed model. Meanwhile, the algorithm is very strict on the camera position; if the camera position is deviated, it will lead to wrong depth prediction. In addition, compared with the original algorithm, the computational speed will be slower, and these problems need to be solved in the future.

In the future, to address the above limitations, we would like to combine other algorithms such as the Superpoint feature point matching algorithm to find the bitmap, and we can complement the sequence image by the neural radiation field of Nerf series. At the same time, we can improve the algorithm to speed up the single-frame processing and add a real-time rendering function to realize a real-time reconstruction and rendering function. We hope to apply the algorithm to more fields of 3D reconstruction.

## Figures and Tables

**Figure 14:**
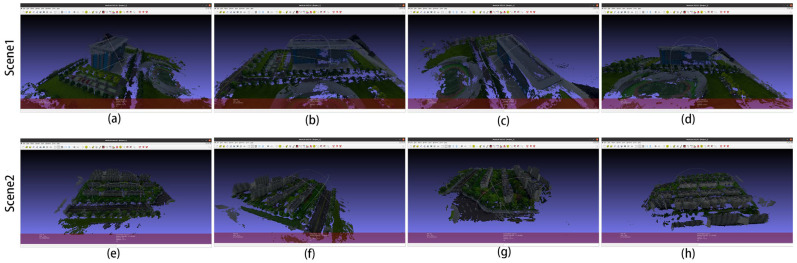
(**a**–**h**) are the three-dimensional reconstruction modeling diagram of the two scenes in Figure 12.

**Table 1:** Comparison of reconstruction quality on DTU dataset.

Method	Acc. (mm)	Comp. (mm)	Overall
MVSNet	0.396	0.527	0.462
R-MVSNet	0.383	0.452	0.417
CasMVSNet	0.3779	0.3645	0.371
PointMVSNet	0.6344	0.6481	0.391
PVA-MVSNet	0.372	0.350	0.361
TransMVSNet	**0.321**	0.289	0.305
Ours	0.3643	**0.225**	**0.295**

Bolded data indicates current best results.

**Table 2:** Comparison of GPU memory usage and runtime on DTU dataset for different input sizes. GPU memory usage and runtime are obtained by running the official evaluation code of baselines on the same machine with an NVIDIA GeForce RTX 3090 laptop graphics card.

Method	Input Size	Depth Map Size	GPU Mem (MB)	Runtime
MVSNet	1600 × 1152	400 × 288	22,511	2.76
R-MVSNet	1600 × 1152	400 × 288	6915	5.09
CasMVSNet	1600 × 1152	640 × 512	5345	0.492
PointMVSNet	1600 × 1152	800 × 576	13,081	3.04
PVA-MVSNet	1600 × 1152	800 × 576	24,870	4.36
TransMVSNet	1600 × 1152	1152 × 864	23,008	0.65
Ours	1600 × 1152	1152 × 864	23,150	1.49

## Data Availability

The DTU is a large dataset containing 128 scenes from controlled laboratory environments modeled with models captured using a structured light scanner (https://roboimagedata.compute.dtu.dk/, accessed on 1 January 2024). The dataset from the leading photogrammetry company Pix4d is publicly available (https://cloud.pix4d.com/demo, accessed on 1 January 2024).

## References

[1] Peng Q., Fei L. Research and Development of Computer Aided Product Innovation Design System. Proceedings of the 2020 5th International Conference on Mechanical, Control and Computer Engineering (ICMCCE).

[2] Yastikli N., Özerdem Ö.Z. (2017). Architectural Heritage Documentation by Using Low Cost Uav with Fisheye Lens: Otag-I Humayun in Istanbul as a Case Study. ISPRS Ann. Photogramm. Remote Sens. Spat. Inf. Sci..

[3] Balci D., Kirimker E.O., Raptis D.A., Gao Y., Kow A.W.C. (2022). Uses of a dedicated 3D reconstruction software with augmented and mixed reality in planning and performing advanced liver surgery and living donor liver transplantation (with videos). Hepatobiliary Pancreat. Dis. Int..

[4] Ann N.Q., Achmad M.S.H., Bayuaji L., Daud M.R., Pebrianti D. Study on 3D scene reconstruction in robot navigation using stereo vision. Proceedings of the 2016 IEEE International Conference on Automatic Control and Intelligent Systems (I2CACIS).

[5] Lu Z., Lv Y., Ai Z., Suo K., Gong X., Wang Y. (2022). Calibration of a Catadioptric System and 3D Reconstruction Based on Surface Structured Light. Sensors.

[6] Langguth F., Sunkavalli K., Hadap S., Goesele M. (2016). Shading-Aware Multi-View Stereo.

[7] Um D., Lee S. (2020). Microscopic Structure from Motion (SfM) for Microscale 3D Surface Reconstruction. Sensors.

[8] Yao Y., Li S., Zhu S., Deng H., Fang T., Quan L. Relative Camera Refinement for Accurate Dense Reconstruction. Proceedings of the 2017 International Conference on 3D Vision (3DV).

[9] Hasson Y., Tekin B., Bogo F., Laptev I., Pollefeys M., Schmid C. Leveraging Photometric Consistency Over Time for Sparsely Supervised Hand-Object Reconstruction. Proceedings of the 2020 IEEE/CVF Conference on Computer Vision and Pattern Recognition (CVPR).

[10] Li S., Xiao X., Guo B., Zhang L. (2020). A Novel OpenMVS-Based Texture Reconstruction Method Based on the Fully Automatic Plane Segmentation for 3D Mesh Models. Remote Sens..

[11] Ruchay A., Dorofeev K., Kalschikov V., Kober A. Accuracy analysis of surface reconstruction from point clouds. Proceedings of the 2020 International Conference on Information Technology and Nanotechnology (ITNT).

[12] Ai L., Xie Z., Yao R., Li L. (2023). R-VPCG: RGB image feature fusion-based virtual point cloud generation for 3D car detection. Displays.

[13] Wu X., Zhou D., Wen P. A MVS based automatic 3D model reconstruction system from turntable image sequence. Proceedings of the 2016 IEEE International Conference on Information and Automation (ICIA).

[14] Yao Y., Luo Z., Li S., Fang T., Quan L. (2018). MVSNet: Depth Inference for Unstructured Multi-View Stereo.

[15] Yao Y., Luo Z., Li S., Shen T., Fang T., Quan L. Recurrent MVSNet for High-Resolution Multi-View Stereo Depth Inference. Proceedings of the 2019 IEEE/CVF Conference on Computer Vision and Pattern Recognition (CVPR).

[16] Chen R., Han S., Xu J., Su H. Point-Based Multi-View Stereo Network. Proceedings of the 2019 IEEE/CVF International Conference on Computer Vision (ICCV).

[17] Yi H., Wei Z., Ding M., Zhang R., Chen Y., Wang G., Tai Y.-W. (2019). Pyramid Multi-view Stereo Net with Self-adaptive View Aggregation. arXiv.

[18] Liu W., Wang J., Qu H., Shen L. (2023). Hierarchical MVSNet with cost volume separation and fusion based on U-shape feature extraction. Multimed. Syst..

[19] Yang J., Mao W., Alvarez J.M., Liu M. (2019). Cost Volume Pyramid Based Depth Inference for Multi-View Stereo. arXiv.

[20] Ding Y., Yuan W., Zhu Q., Zhang H., Liu X., Wang Y., Liu X. TransMVSNet: Global Context-aware Multi-view Stereo Network with Transformers. Proceedings of the 2022 IEEE/CVF Conference on Computer Vision and Pattern Recognition (CVPR).

[21] Wu Z.-Z., Zou C., Wang Y., Tan M., Weise T. (2021). Rotation-aware representation learning for remote sensing image retrieval. Inf. Sci..

[22] Sun L., Liu B., Tao J., Lian Z. Multimodal Cross- and Self-Attention Network for Speech Emotion Recognition. Proceedings of the ICASSP 2021—2021 IEEE International Conference on Acoustics, Speech and Signal Processing (ICASSP).

[23] Xu C., Qi Y., Wang Y., Lou M., Pi J., Ma Y. (2022). ARF-Net: An Adaptive Receptive Field Network for breast mass segmentation in whole mammograms and ultrasound images. Biomed. Signal Process. Control.

[24] Chen X., Li Q., Li R., Cai X., Wei J., Zhao H. (2023). UAV Network Path Planning and Optimization Using a Vehicle Routing Model. Remote Sens..

[25] Du M., Li H., Roshanianfard A. (2022). Design and Experimental Study on an Innovative UAV-LiDAR Topographic Mapping System for Precision Land Levelling. Drones.

[26] Pan L., Gu L., Ren R., Yang S. (2020). Land Cover Classification Based on Machine Learning Using UAV Multi-Spectral Images.

[27] Eskandari R., Mahdianpari M., Mohammadimanesh F., Salehi B., Brisco B., Homayouni S. (2020). Meta-analysis of Unmanned Aerial Vehicle (UAV) Imagery for Agro-environmental Monitoring Using Machine Learning and Statistical Models. Remote Sens..

[28] Goulas D., Georgopoulos A., Sarakenos A., Paraschou C. (2013). 3D Mapping from High Resolution Satellite Images.

[29] Fan Y.-W., Zhu W.-J., Ban S.-H. (2019). Mimic Geographic Information System. E3S Web of Conferences, Proceedings of 2018 International Seminar on Food Safety and Environmental Engineering (FSEE 2018), Guangzhou, China, 30 November–2 December 2018.

[30] Bittmann F., Bungenstock F., Wehrmann A. (2022). Drowned palaeo-landscapes: Archaeological and geoscientific research at the southern North Sea coast. Neth. J. Geosci..

[31] Li Z., Li E., Xu T., Samat A., Liu W. (2023). Feature Alignment FPN for Oriented Object Detection in Remote Sensing Images. IEEE Geosci. Remote Sens. Lett..

[32] Yang G., Lei J., Zhu Z., Cheng S., Feng Z., Liang R. (2023). AFPN: Asymptotic Feature Pyramid Network for Object Detection. arXiv.

[33] Feng X., Wang T., Yang X., Zhang M., Guo W., Wang W. (2023). ConvWin-UNet: UNet-like hierarchical vision Transformer combined with convolution for medical image segmentation. Math. Biosci. Eng..

[34] Rajeh S., Savonnet M., Leclercq E., Cherifi H. (2022). Modularity-Based Backbone Extraction in Weighted Complex Networks.

[35] Qiu M., Huang L., Tang B.-H. (2022). ASFF-YOLOv5: Multielement Detection Method for Road Traffic in UAV Images Based on Multiscale Feature Fusion. Remote Sens..

[36] Elfwing S., Uchibe E., Doya K. (2018). Sigmoid-weighted linear units for neural network function approximation in reinforcement learning. Neural Netw..

[37] Zhang D.-Y., Zhang W., Cheng T., Zhou X.-G., Yan Z., Wu Y., Zhang G., Yang X. (2023). Detection of wheat scab fungus spores utilizing the Yolov5-ECA-ASFF network structure. Comput. Electron. Agric..

[38] Wang B., Teng Y., Lau V., Han Z. (2023). CCA-Net: A Lightweight Network Using Criss-Cross Attention for CSI Feedback. IEEE Commun. Lett..

[39] Zheng T., Wang Q., Shen Y., Lin X. (2022). Gradient rectified parameter unit of the fully connected layer in convolutional neural networks. Knowl.-Based Syst..

[40] Aanæs H., Jensen R.R., Vogiatzis G., Tola E., Dahl A.B. (2016). Large-Scale Data for Multiple-View Stereopsis. Int. J. Comput. Vis..

[41] Caputo T., Bellucci Sessa E., Marotta E., Caputo A., Belviso P., Avvisati G., Peluso R., Carandente A. (2023). Estimation of the Uncertainties Introduced in Thermal Map Mosaic: A Case of Study with PIX4D Mapper Software. Remote Sens..

[42] Cignoni P., Callieri M., Corsini M., Dellepiane M., Ganovelli F., Ranzuglia G. MeshLab: An Open-Source Mesh Processing Tool. Proceedings of the European Interdisciplinary Cybersecurity Conference.

[43] Zhao L., Zhang H., Mbachu J. (2023). Multi-Sensor Data Fusion for 3D Reconstruction of Complex Structures: A Case Study on a Real High Formwork Project. Remote Sens..

